# Bone quality in pycnodysostosis: micropetrosis, locally distorted osteocyte lacuno-canalicular network, and heterogenous mineralization pattern in an adult female patient with multiple fractures

**DOI:** 10.1093/jbmrpl/ziaf015

**Published:** 2025-01-23

**Authors:** Nadja Fratzl-Zelman, Stéphane Blouin, Uwe Kornak, Markus A Hartmann, Andreas A Kurth, Jochen Zwerina

**Affiliations:** Ludwig Boltzmann Institute of Osteology at the Hanusch Hospital of OEGK and AUVA Trauma Center Meidling, 1st Medical Department Hanusch Hospital, 1140 Vienna, Austria; Vienna Bone and Growth Center, Vienna, Austria; Ludwig Boltzmann Institute of Osteology at the Hanusch Hospital of OEGK and AUVA Trauma Center Meidling, 1st Medical Department Hanusch Hospital, 1140 Vienna, Austria; Vienna Bone and Growth Center, Vienna, Austria; Institute of Human Genetics, University Medical Center Göttingen, 37075 Göttingen, Germany; Ludwig Boltzmann Institute of Osteology at the Hanusch Hospital of OEGK and AUVA Trauma Center Meidling, 1st Medical Department Hanusch Hospital, 1140 Vienna, Austria; Vienna Bone and Growth Center, Vienna, Austria; Center for Orthopaedics and Trauma Surgery, Marienhaus Klinikum Mainz, 55131 Mainz, Germany; Ludwig Boltzmann Institute of Osteology at the Hanusch Hospital of OEGK and AUVA Trauma Center Meidling, 1st Medical Department Hanusch Hospital, 1140 Vienna, Austria; Vienna Bone and Growth Center, Vienna, Austria

**Keywords:** *CTSK*, osteoclast, bone histology, osteocyte lacunae, osteocyte lacuno-canalicular network

## Abstract

Pycnodysostosis is a very rare skeletal dysplasia caused by biallelic loss-of-function mutations in cathepsin K, a proteolytic enzyme highly expressed by osteoclasts. Deficiency of cathepsin K impairs bone resorption and further bone remodeling leading to progressive osteosclerosis and bone fragility. Moreover, cathepsin K is also expressed by mature osteocytes. Whether the density, size, and viability of osteocytes and the osteocyte lacuno-canalicular network (OLCN) are also altered, thereby impacting bone quality in pycnodysostosis, has not been explored. We used light microscopy, quantitative backscattered electron imaging, and confocal laser scanning microscopy to examine bone material obtained from a 57-yr-old female patient during surgical correction after femoral head fracture. The cortex consisted of a compact shell of multilayered collagen fibrils oriented in parallel to the periosteum, reflecting vigorous primary bone apposition, multiple osteons with concentrically ordered lamellae, and scattered patches of woven bone. The trabecular area was very dense with trabecular bone volume, varying locally from 30.3% to 67.4%. The bone matrix was overmineralized (average calcium content: +7.5% versus reference values, with a 5-fold increase of highly mineralized areas >27 weight % calcium). Numerous multinucleated osteoclasts and fringes of demineralized matrix were viewed on bone surfaces. The density (number/mm^2^: 193 to 223) and area (20 μm^2^) of the osteocyte lacunae and their canalicular length (0.05 μm/μm^3^ bone volume) were within normal range. However, numerous bone packets exhibited (hyper)mineralized osteocyte lacunas (micropetrosis) resulting in a locally disrupted OLCN. In summary, our data indicate that in pycnodysostosis not only osteoclast function is impaired but also osteocyte viability is decreased, leading to micropetrosis, distorted OLCN, and heterogenous mineralization pattern. Thus, osteoclasts and osteocytes both contribute to reduce bone quality. However, the presence of a dense osteocyte network in large areas of the sample indicates that cathepsin K is not essential for the formation of the OLCN.

## Graphical Abstract

**Graphical Abstract:**
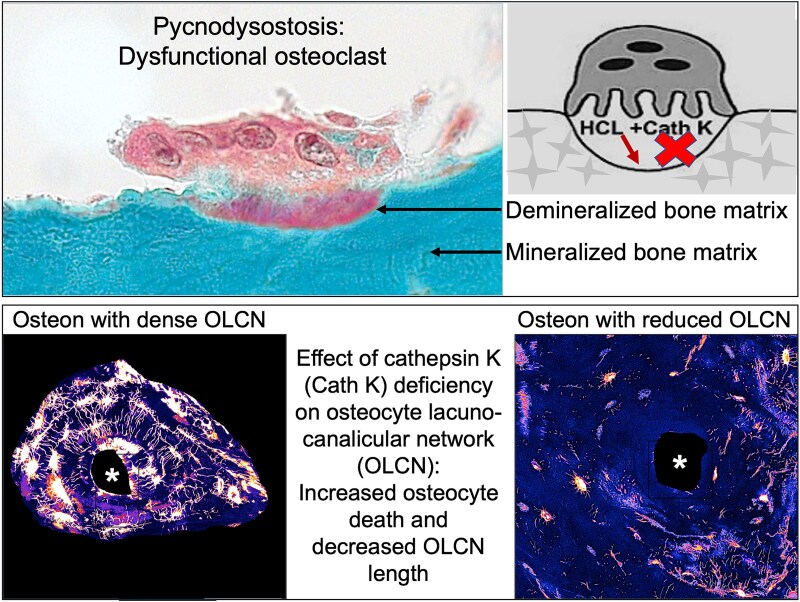

## Introduction

Pycnodysostosis (OMIM 265800) is an ultra-rare skeletal dysplasia with an estimated prevalence of 1-3/1 000 000.^1^ The disorder is caused by biallelic loss-of-function mutations in *CTSK* the gene encoding cathepsin K, a lysosomal cysteine protease highly expressed by osteoclasts.^2^ Patients present with short stature, craniofacial and oro-dental abnormalities, acro-osteolysis of the distal phalange, progressive osteosclerosis, and recurrent fractures.^1^^,^^3–5^ During bone remodeling, cathepsin K cleaves collagen type I and type II and absence of the enzyme leads to dysfunctional osteoclasts that differentiate and maintain their demineralizing capacity but are unable to further digest the collagenous matrix. As a result, demineralized collagen accumulates either on bone surfaces or might be engulfed in lysosomal vacuoles of cathepsin K-deficient osteoclasts.^6^^,^^7^ Collagen leftover might be partially degraded by other proteases that are upregulated as a compensatory mechanism to counteract the lack of cathepsin K and bone turnover of patients with pycnodysostosis is maintained to a certain extent^8–11^ or even increased.^12^ However, transiliac biopsy analyses from young patients revealed not only elevated bone volume and increased bone matrix mineralization, but also a profound deterioration of the bone matrix with highly disordered lamellar organization and poorly aligned mineral particles.^7^ In line with these observations, an abnormal bone microstructure consisting of large amounts of poorly organized woven bone was noticed in cathepsin K-deficient mice.^13^ Thus, these findings indicate that the lack of cathepsin K in patients with pycnodysostosis impacts not only bone remodeling but also bone quality, which probably cannot be attributed solely to disrupted osteoclast activity.

Little attention has been given to the fact that cathepsin K is also expressed by mature osteocytes within the mineralized bone matrix and has a critical role in osteoclast independent bone matrix remodeling processes.^14^^,^^15^ Osteocytes with their multiple dendritic processes residing in lacunae and canaliculi, respectively, are not only the cellular effectors and controllers of bone remodeling but further do resorb their surrounding matrix to provide calcium to the body in situations of higher physiological demand, and under pathological conditions of calcium deficiency (osteocytic osteolysis).^14–16^ Moreover, osteocytes are mechanosensitive cells and remodeling of the osteocytic perilacunar/canalicular matrix allows the skeleton to adapt continuously to mechanical loading.^14^ Conversely, the mechano-response of bone is closely related to the osteocyte lacuno-canalicular network (OLCN)^17^ architecture and the latter is linked to the orientation of the surrounding collagen matrix.^18^^,^^19^ In accordance, a recent study showed that abrogation of perilacunar/canalicular remodeling through the targeted deletion in osteocytes of the transcriptional regulators Yes-associated protein (YAP) and the transcriptional co-activator with PDZ-binding motif (TAZ) resulted not only in decreased expression of cathepsin K (and other matrix proteases) in osteocytes, but furthermore also in reduced density of the OLCN, disordered collagen matrix organization and impaired mechanical properties.^20^ Whether beyond osteoclast dysfunction also the OLCN is altered in patients with pycnodysostosis and thereby contribute to bone fragility has not yet been explored.

Here we investigated bone histology, bone mineralization density distribution (BMDD), osteocyte lacunae characteristics, and OLCN of a female adult patient who underwent surgical correction after femoral head fracture. We show that the OLCN is ordered and forms a dense network as in healthy individuals but is locally disrupted by accumulation of occluded osteocyte lacunae (micropetrosis).

## Materials and methods

### Bone samples

We examined discarded bone material from the femoral head from a 57-yr-old female patient who underwent surgical correction. Written informed consent for data publication was obtained from the patient.

Because OLCN reference data from healthy adults are not yet available, we further analyzed OLCN characteristics in the trabecular regions in iliac crest autopsy samples taken from the standard biopsy site (2 cm from spina iliaca superior anterior and 2 cm from the crest) from 2 donors without any known bone disease. Both donors were female with an age of 56.9 and 61.7 yr, respectively. These samples were provided from the Unit of Forensic Gerontology, Medical University of Vienna with ethical approval number EK No. 1757/2013.

### Determination of the *CTSK* mutation

DNA was isolated from an EDTA blood sample. A custom designed SureSelect XT gene panel (Agilent) was used to enrich the coding exons of genes associated with skeletal dysplasias, dysostoses, or connective tissue diseases (skeletal disease-associated genome panel; sDAG) as described before.^21^ After next-generation sequencing (NGS) on a NextSeq device (Illumina), alignment and variant calling using standard pipelines genetic variants were prioritized according to frequency, pathogenicity, and phenotype relevance using PhenIX,^22^ GeneTalk,^23^ and MutationTaster.^24^ The classification of the detected genetic variants was performed according to the American College of Medical Genetics and Genomics (ACMG).^25^

### Sample preparation

The bone sample was fixed in 70% ethanol, dehydrated in graded series of alcohol, stained with rhodamine 6G for visualization of the OLCN, and embedded in polymethyl-methacrylate (PMMA).^18^

### Light microscopy

Three to four micrometer thin sections were cut from the sample block using a hard tissue microtome (Leica SM2500). Subsequently, the sections were de-plasticized using 2-methoxyethylacetate and stained either with modified Goldner’s Trichrome or with Giemsa. The stained sections were investigated using a light microscope (Axiophot, Zeiss) equipped with a digital camera (AxioCam HRc, Zeiss).

### Quantitative backscattered electron imaging

For the quantitative backscattered electron imaging (qBEI) analyses the sectioned bone surfaces from the residual PMMA-embedded sample block was ground with sandpaper with increasing grit-number, polished with diamond grains (size down to 1 μm) and carbon-coated by vacuum-evaporation (Agar SEM carbon-coater; Agar Scientific Stansted).

Backscattered electron images of the bone sample were collected using a field emission scanning electron microscope (FESEM, SEM SUPRA 40, Zeiss) at 20 kV, 10 mm working distance, and 270-320 pA sample current with 1.76 μm spatial resolution. A high purity aluminum/carbon reference sample was used for calibration to compare between measurements.^26^

### Bone mineralization density distribution

Gray level histograms were deduced and transformed into calcium weight percent to evaluate BMDD^26^ and the histomorphometric parameter of mineralized (md.) trabecular bone volume (BV/TV) according to the standardized terminology established by the working group of the American Society for Bone and Mineral Research.^27^ The BMDD is characterized by 5 parameters: CaMean (weight % Ca) is the weighted average calcium concentration of the mineralized tissue area; CaPeak (weight % Ca) indicating the most frequently occurring calcium concentration in the sample (peak position of the curve); CaWidth (Δ weight % Ca), width at the half maximum reflecting the heterogeneity of mineralization; CaLow (% bone area), the fraction of lowly mineralized bone tissue, defined as the percentage of bone area that is mineralized below the fifth percentile of the reference BMDD curve (% bone area mineralized < 18.20 weight % Ca); and CaHigh (% bone area), the fraction of highly mineralized bone tissue, defined as the percentage of bone area that is mineralized above the 95th percentile of the reference BMDD curve (% bone area mineralized > 26.86 weight % Ca). The obtained values were compared with published BMDD reference data from iliac crest from 25 healthy adults.^26^

### Osteocyte lacunae sections characteristics

Osteocyte lacunae section (OLS) analyses were performed on 0.88 μm/pixel grey-level images of the 3 regions using a custom-made macro in ImageJ software (version 1.52; NI) by setting a threshold based on a fixed grey-level (5.2 weight % calcium) to discriminate osteocyte lacunae from surrounding mineralized bone matrix and a size threshold between 5 and 200 μm^2^ to distinguish between OLS from larger channels, as described previously.^28^

The OLS are characterized by 5 parameters: OLS-density (OLS-number/mm^2^): the number of OLS per mineralized bone matrix area; OLS-porosity: (%) OLS total area/(mineralized bone matrix area + OLS total area); OLS-area (μm^2^): median value of the OLS areas per sample; OLS-perimeter (μm): median value of the OLS perimeter per sample; and OLS-aspect ratio: median value of the OLS-aspect ratio per sample. The OLS-aspect ratio is a measure for the shape of the OLS. It is given by the ratio of the long to the short half-axis of a fitted ellipse to the section. A value of 1 indicates a perfect circle, while increasing values indicate an increasing elongated shape. OLS with aspect ratio values >10 were excluded from the analysis. The obtained values were compared with published OLS reference data from iliac crest from healthy adults.^28^

### Micropetrosis

To determine the extent of micropetrosis, we evaluated the number of mineralized osteocyte lacunae sections (md.OLS) on the OLS images, appearing as hypermineralized areas compared with the surrounding bone matrix. We applied a semiautomatic method to detect the md.OLS using a custom-made macro in ImageJ (version 1.52; NI) based on a fixed threshold of 31.02 weight % Ca to discriminate between mineralized bone and md.OLS, a size range between 10 and 200 μm^2^ and circularity range between 0.35 and 1 (shape descriptor provided by ImageJ), as described previously.^28^ The number of md.OLS per total bone area (md.OLS-density) was evaluated. The obtained values were compared with published md.OLS reference data from iliac crest from healthy adults.^28^

### Quantitative energy dispersive X-ray spectrometry of “macropetrosis”

Throughout the sample we observed large, highly mineralized inclusions (dystrophic calcifications, denoted as “macropetrosis”. The elemental composition of these inclusions and of adjacent “normal” mineralized regions was determined by quantitative energy dispersive X-ray spectrometry (EDX) using a Zeiss Supra 40 field emission scanning electron microscope (FESEM) (Zeiss) equipped with a large-area (80 mm^2^) EDS Silicon Drift Detector (X-Max, Oxford Instrument). The analysis of the generated EDX spectra was carried out using Oxford INCA software. The FESEM was operated at 10 kV with a probe current of about 1.5 nA at 10 mm working distance and an aperture size of 60 μm. A resolution of about 28.65 nm pixel size was used. Custom ROIs were continuously scanned with a speed of 3.2 μs/pixel. All spectra were acquired with an acquisition time of 30 s, a 0-10 keV spectrum range with 2000 channels. The systems energy canal positions were fine-tuned by acquisition of a spectrum from a Ni sample of high purity. Only homogeneous bone areas (ROIs) excluding osteocyte lacunae were measured. Ca, P, Mg, Na, O, and C atomic percentage were determined with the processing option of carbon by difference.

### Confocal laser scanning microscopy for evaluation of OLCN

To assess the OLCN the rhodamine-stained sample was measured on a confocal laser scanning microscope (Leica TCS SP5, Leica). Measurements were performed with an oil immersion objective (HCX PL APO CS40.0x1.25) having a lateral resolution of 0.38 μm per pixel. Fluorescence of rhodamine was excited with a HeNe-Laser at a wavelength of 543 nm. The fluorescent signal was collected using an airy 1 pinhole (67.93 μm) in a detection window between 553 and 705 nm. Image stacks were recorded with a depth resolution of 0.25 μm. Properties of the OLCN were calculated from obtained image stacks using the Tool for Image and Network Analysis (TINA, https://gitlab.mpikg.mpg.de/rummler/TINA). In short, TINA is a summary of python scripts to binarize, segment, and skeletonize fluorescent images as well as to build a 3D network from the obtained data and tools for its subsequent analysis. Binarization and segmentation is done using difference of Gaussians with different kernel size to obtain and discriminate between canaliculi and larger structures like lacunae and vascular channels. Resulting binarized images are skeletonized and a 3D network is built in Networkx 3.2.1. The main parameter of this analysis is the network density, that is, the total canalicular length per volume measured in μm/μm^3^. The obtained values were compared with OLCN characteristics from the trabecular regions in iliac crest autopsy samples obtained from 2 donors as described above.

## Results

### Clinical report of the patient

Our patient was born in 1965 by forceps delivery with normal weight and stature. At 2 yr of age, growth retardation was noted and injury of pituitary gland secondary to forceps delivery was hypothesized. She was treated with (unknown) hormones until 6 yr of age. During childhood she occasionally suffered from sudden seizures and was several times unconscious when bumping her head. Electroencephalography check-ups showed no abnormalities. At the age of 6 yr, she was diagnosed with dwarfism due to damage of the pituitary gland. Her adult height is 134 cm (<1 percentile).

The patient attended regular primary and secondary schools, had normal daily life activities with walking, running, cycling, and skating like her classmates. Nonetheless, during that time she suffered from 3 tibial and 2 femoral fractures that were subsequently treated conservatively with plaster cast immobilization. Defective and delayed bone healing was noted. At the age of 23 yr, she experienced a first femoral neck fracture, requiring hospitalization for an extension therapy followed by a pelvic cast.

In the following years she suffered from recurrent fractures of the tibiae and fibulae that were always treated conservatively. She underwent a first surgical intervention with 2 plates at the age of 38 yr after refracture of the tibia and fibula following a bicycle fall. These implants are still in place and functional today. In the following years she suffered from numerous rib fractures that occurred spontaneously or after coughing and sneezing. At the age of 45 yr, after a fracture of the left femur, a large gap was detected, corrective surgery was performed but no bone healing was noted. She was then referred to the Center for Muscle and Bone Diseases to the Charité Berlin, where open fontanelles and generalized osteosclerosis were diagnosed. First DXA measurements at the age of 46 yr showed BMD Z-score at L1-L4: +4.0 and at the proximal femoral bone: +5.3. An underlying genetic bone fragility disorder was suspected, and genetic testing was initiated. In the next months, she underwent 5 surgical interventions to restore bone defects. In 2020 she sustained again a femoral fracture requiring series of surgical reconstructions. However, in fall 2022 a reconstruction with a megaprothesis (including a knee prothesis due to severe osteoarthritis) was mandatory. During surgery, femoral head tissue was discarded and used for the present analyses. Blood count test prior surgical intervention showed that all parameters were within normal range, except the number of erythrocytes, which was decreased (2.6 million/mm^3^, lower limit of normal: 3.9 million/mm^3^). The evaluated biochemical variables (alkaline phosphatase, PTH, vitamin D, calcium, and phosphorus) were also within normal limits (see Table S1).

### Molecular diagnosis of pycnodysostosis by gene panel sequencing

**Figure 1:**
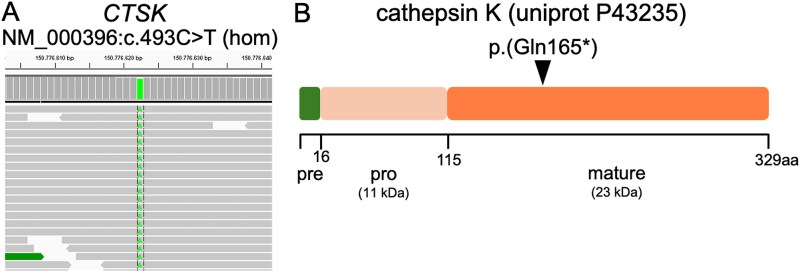
(A) Integrated genome viewer representation of the homozygous, pathogenic CTSK variant c.493C>T. (B) Position of the truncating mutation p.(Gln165^*^) within the proteolytically active part of the cathepsin K protein. Cathepsin K is subdivided into a 16 aa pre-, a 99 aa pro-, and a 214 aa mature protein, which is active after cleavage of the pre- and pro-domain.

Genetic variants obtained by gene panel sequencing were prioritized according to the available clinical information (osteosclerosis, recurrent fractures). Top ranking was the homozygous variant c.493C>T in the gene *CSTK*, which leads to a premature stop codon p.(Gln165^*^) strongly truncating the gene product cathepsin K (Figure 1). This variant is neither mentioned in gnomAD nor ClinVar but is clearly pathogenic using the ACMG criteria. Criterium PVS1 (8 points) can be used because the variant leads to a loss of function and pycnodysostosis is known to be caused by such variants,^1–3^ PM2_SUP (1 point) is valid since the variant is not found in the general population, PP4 (1 point) can be chosen because genotype and phenotype match perfectly. In summary, 10 points allow to classify the variant as pathogenic. At the same codon the missense variant p.(Gln165Arg) had been described previously.^29^

### Bone sample shows high trabecular density and high bone mineral content

**Figure 2:**
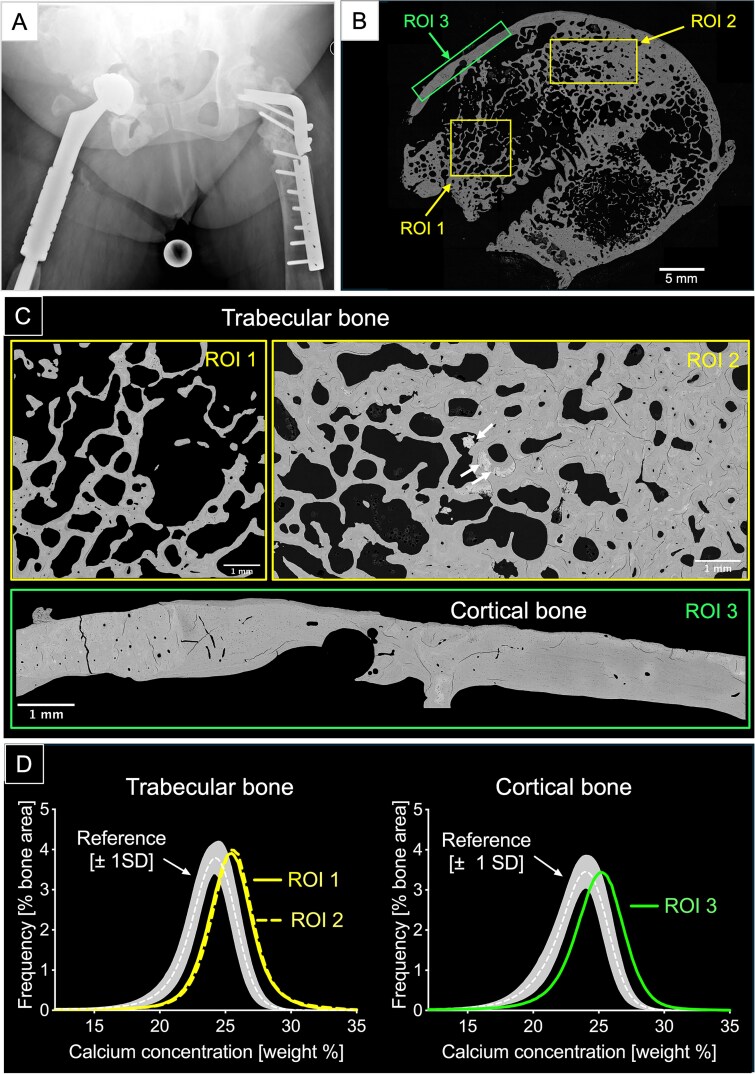
(A) Proximal femur radiography prior corrective surgery. (B) Overview of the entire sectional area of the bone sample obtained post-surgery, imaged by quantitative backscattered electron imaging (qBEI) and showing 3 ROIs that were analyzed. ROI 1: spongy trabecular like area (BV/TV: 30.3%); ROI 2: more compact trabecular like area (BV/TV: 67.4%); ROI 3: area within the cortical bone. Scalebar = 5 mm. (C) Backscattered electron microscopy images of the 3 ROIs at larger magnification: the white arrows point toward highly mineralized inclusions (“macropetrosis,” see also Figure 3E). Note that only mineralized matrix in grey is viewed by qBEI. Lower mineralized areas appear dark gray, higher mineralized areas are bright. The embedding medium, the bone marrow, the unmineralized matrix, and the unmineralized osteocyte lacunae within the mineralized bone appear black. Scalebars = 1 mm. (D) Bone mineralization density distribution (BMDD): all 3 ROIs show abnormally high bone matrix mineralization: all 3 curves are shifted to the right; we compared the reference BMDD curve of heathy individuals^26^ (all BMDD parameters are compiled in Table 1).

The femoral bone sample obtained during surgical intervention (Figure 2A) consisted of an area of trabecular bone surrounded by a cortical shell of compact bone (Figure 2B). We evaluated qBEI images from 3 different sites of the bone specimen: a more spongy trabecular-like region (ROI 1), a more compact trabecular region (ROI 2) and a region within the cortex (ROI 3) (Figure 2B and C). The trabecular volume BV/TV was elevated, varying locally from 30.3% in ROI 1 to 67.4% in ROI 2. In both trabecular regions as well as in the cortical bone (ROI 3), the bone matrix was highly mineralized and did not substantially differ between the 3 regions (average calcium content: CaMean +7.5%, absolute increase above reference mean value) and a 5-fold increase of highly mineralized areas (CaHigh) versus reference values^26^ (Figure 2D, Table 1).

**Table 1:** qBEI results for bone mineralization density distribution (BMDD).

**BMDD parameters**	**Trabecular bone** **ROI 1**	**Trabecular bone** **ROI 2**	**Reference healthy adults** **trabecular bone** ^**24**^	**Cortical bone** **ROI 3**	**Reference healthy adults** **cortical bone** ^**24**^
**CaMean (weight % Ca)**	24.86	25.14	23.26 (0.58)	24.84	22.96 (0.57)
**CaPeak (weight % Ca)**	25.30	25.48	24.1 (0.51)	25.13	23.89 (0.54)
**CaWidth (Δ weight % Ca)**	3.81	3.81	3.87 (0.28)	4.16	4.09 (0.41)
**CaLow (% bone area)**	3.36	2.93	4.61 [3.95; 5.57]	3.12	5.62 (1.43)
**CaHigh (% bone area)**	20.67	23.17	4.70 (2.84)	18.79	3.70 [2.12; 4.80]

Abbreviation: qBEI, quantitative backscattered electron imaging.

### Bone histology shows dysfunctional osteoclasts, marrow fibrosis, and large areas of woven bone

**Figure 3:**
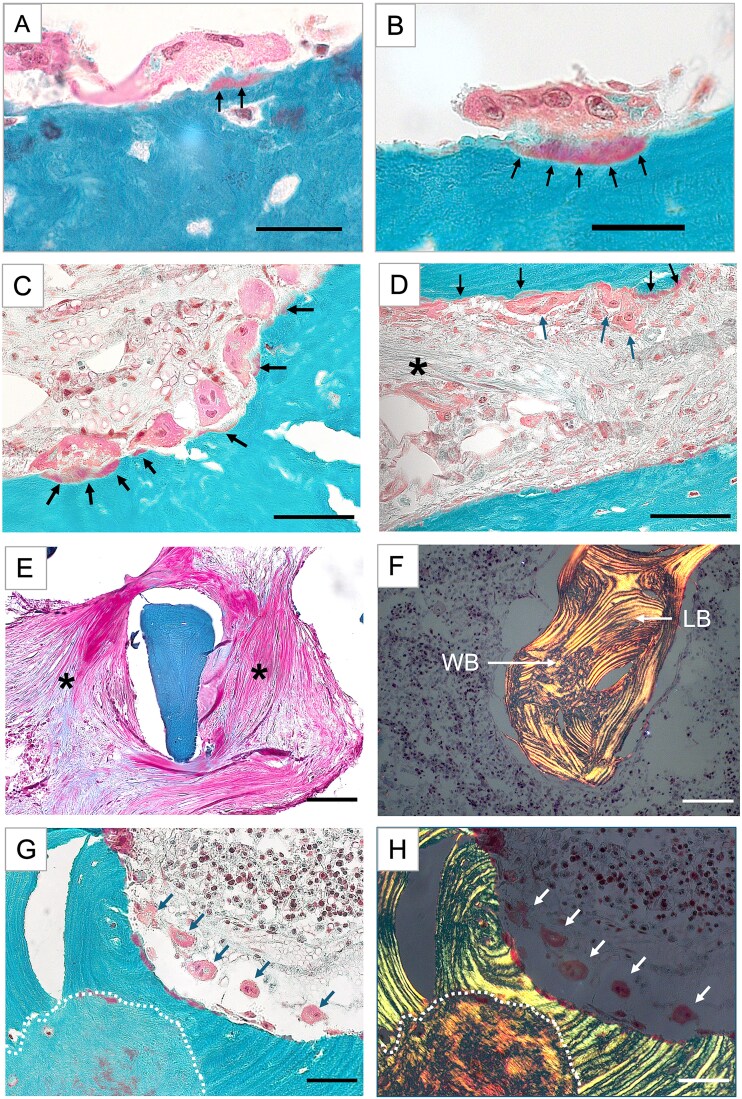
Distinctive histological features in the bone sample from the patient with pycnodysostosis. Goldner stained sections, trabecular-like areas (ROI 1 and ROI 2). Mineralized areas stain green, unmineralized areas stain red. (A and B) Pycnodysostotic osteoclasts exhibiting many nuclei and vacuoles (lysosomes). The most characteristic feature in pycnodysostosis are the red stained lacunae below the osteoclasts on the bone surface (black arrows), demonstrating that the bone matrix is demineralized but the collagenous matrix is not removed. Scalebars = 25 μm. (C and D) Areas with numerous adjacent osteoclasts (blue arrows). Note that many demineralized resorption lacunae appear rather flat (black arrows). Note also the presence of bone marrow fibrosis (black asterisks). Scalebars = 50 μm. (E) Isolated trabecular feature (green) surrounded by very compact bone marrow fibrosis (black asterisks). Scalebar = 100 μm. (F) Trabecular feature viewed under polarized light showing areas with parallel lamellar bone (LB) interrupted by patches of disordered woven bone (WB). Scalebar = 100 μm. (G and H) Detail from ROI 2. Identical bone area viewed in bright field (G) and under polarized light (H) showing a large island of highly disordered woven bone (delineated by the white broken line) within an area of parallel lamellar bone. The arrows point toward osteoclasts that are detached from the bone surface. Scalebars = 50 μm.

Trabecular bone: light microscopic examination of Goldner stained sections revealed typical features of pycnodysostotic osteoclasts: large multinucleated cells with numerous intracellular vacuoles, located adjacent to areas of demineralized collagenous matrix on the bone surface (red staining). The resorption cavities appeared rather flat (Figure 3A and B). In accordance with observations from other forms of osteopetrosis due to dysfunction of the differentiated osteoclasts,^30^ numerous adjacent osteoclasts were viewed on trabecular surfaces (Figure 3C and D). Some areas of the bone marrow exhibited intense fibrosis (Figure 3D and E). The mineralized bone matrix consisted of a mixture of ordered lamellar bone and disordered woven bone (Figure 3F-H).

**Figure 4:**
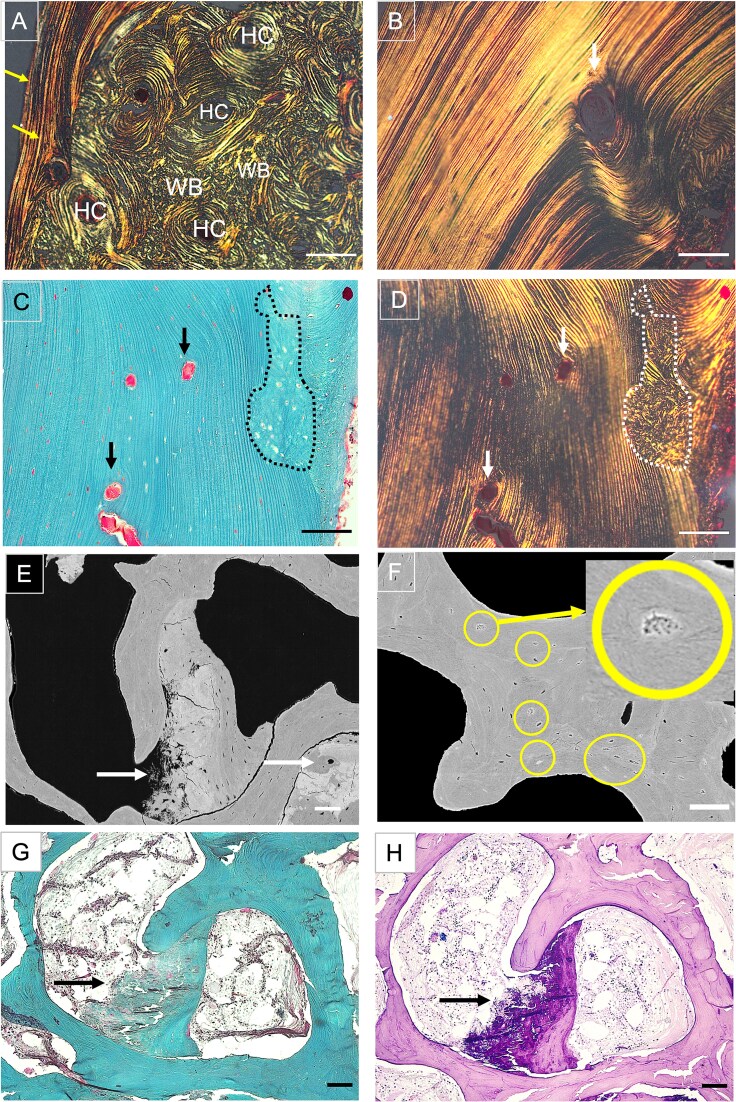
(A-D) Distinctive histological features in the bone sample from the patient with pycnodysostosis. Goldner stained sections, cortical area (ROI 3). (A) Two distinct regions can be observed under polarized light: a shell of primary (not remodeled) bone, formed by periosteal apposition, consisting of parallelly arranged lamellae along the periosteum (yellow arrows) and intra-cortically, osteonal remodeled bone, characterized by concentrically arranged bone lamellae around a central channel (Haversian channels, HC). Between the osteons, patches of highly disordered woven bone are viewed (WB). Scalebar = 100 μm. (B) Detail from the area of primary lamellar bone viewed under polarized light. Note that bone lamellae are arranged in parallel over a long distance and this structure is here interrupted by a newly formed vascular channel (white arrow). Scalebar = 100 μm. (C and D) Identical bone area viewed in bright field (C) and under polarized light (D) showing also within the parallel lamellar bone, inclusions of highly disordered woven bone (black broken line in C, white broken line in D). The arrows point toward vascular channels. Scalebars = 100 μm. (E-F) Macropetrosis (E) and micropetrosis (F) detected on qBEI images: (E) Macropetrosis: irregularly formed calcified patched are viewed between trabecular features or trapped within the bone matrix. They appear bright on the backscattered image and are therefore highly mineralized. These inclusions differ in size and shape from occluded osteocyte lacunae, which are also highly mineralized; micropetrosis, in (F) within the yellow circles, insert: micropetrotic osteocyte lacuna viewed at larger magnification. Scalebars = 100 μm. (G and H) Same region with macropetrosis than in E viewed in a Goldner stained section (G) and with Giemsa staining (H). Note that the irregularly shaped structure (see arrows in both images) is hardly visible in Goldner stained sections, since Goldner stains mineralized bone in green irrespective of the mineral content. In contrast with Giemsa the structure stains dark purple (in contrast to pale pink typical for lamellar bone) indicating a distinct dystrophic calcification. Scalebars = 100 μm.

Cortical bone: the cortex of the sample consisted of a compact shell of parallelly ordered fibrils, reflecting vigorous periosteal bone apposition and multiple Haversian channels with concentrically ordered lamellae typical for osteonal remodeling (Figure 4A-D). However, the area between the concentric lamellae was occupied by woven bone (Figure 4A). Also irregularly shaped patches of woven bone often interrupted the parallelly arranged lamellae below the periosteum (Figure 4C and D**).**

### Elevated micro- and macropetrosis

Throughout the sample, the backscattered electron microscopy images revealed large hypermineralized inclusions as indicated by white arrows in Figure 2C. In contrast to “micropetrosis,” that is, mineralized osteocyte lacunae that were also found to be highly increased in numerous bone packets throughout the sample (Figure 4F, Table 2), we termed the observed large inclusions “macropetrosis.” (Figure 4E) These inclusions could not be easily identified on histological sections, since Goldner stains mineralized bone matrix irrespectively of the mineral content. Based on the irregular structure of these inclusions, their abnormal localization within the bone matrix or between bone struts, and by comparison with identical areas on Giemsa-stained sections (Figure 4G and H), these areas could be further characterized. The mineral nature of these structures was confirmed by EDX analyses, showing that the calcium to phosphate ratio was 1.7, the typical ratio for hydroxyapatite, and did not differ between areas of macropetrosis and adjacent mineralized bone tissue (see Table S2). However, the carbon content was considerably reduced, indicating lower organic (cellular) components.

**Table 2:** qBEI results for osteocyte lacunar sections (OLS).

**ROI** **(number of analyzed OLS)**	**Trabecular bone** **ROI 1** **(1068 OLS)**	**Trabecular bone** **ROI 2** **(1981 OLS)**	**Reference trabecular bone** **adult 57 yr** ^**26**^	**Cortical bone** **ROI 3** **(1274 OLS)**	**Reference** **cortical bone** **adult 57 yr** ^**26**^
**OLS-porosity (%)**	0.5	0.4	0.46 ± 0.07	0.5	0.47 ± 0.08
**OLS-density (nb/mm^2^)**	212.4	192.5	187.46 ± 23.58	223.1	215.97 ± 44.37
**OLS-area (μm^2^)**	21.0	17.1	20.73 ± 1.9	21.0	18.44 ± 2.62
**OLS-perimeter (μm)**	20.8	19.3	20.41 ± 0.94	22.0	18.89 ± 1.67
**OLS-aspect ratio**	2.5	2.3	2.87 ± 0.18	3.1	2.51 ± 0.24
**Md OLS-density (nb/mm^2^)** **“Micropetrosis”**	**27.19**	**8.88**	1.88 ± 1.52	**18.13**	3.22 ± 2.41

Note the high number of micropetrotic osteocyte lacunae in patient’s bone (in bold).

Abbreviation: qBEI, quantitative backscattered electron imaging.

### Osteocyte lacunae shape reflects the surrounding bone tissue organization

**Figure 5:**
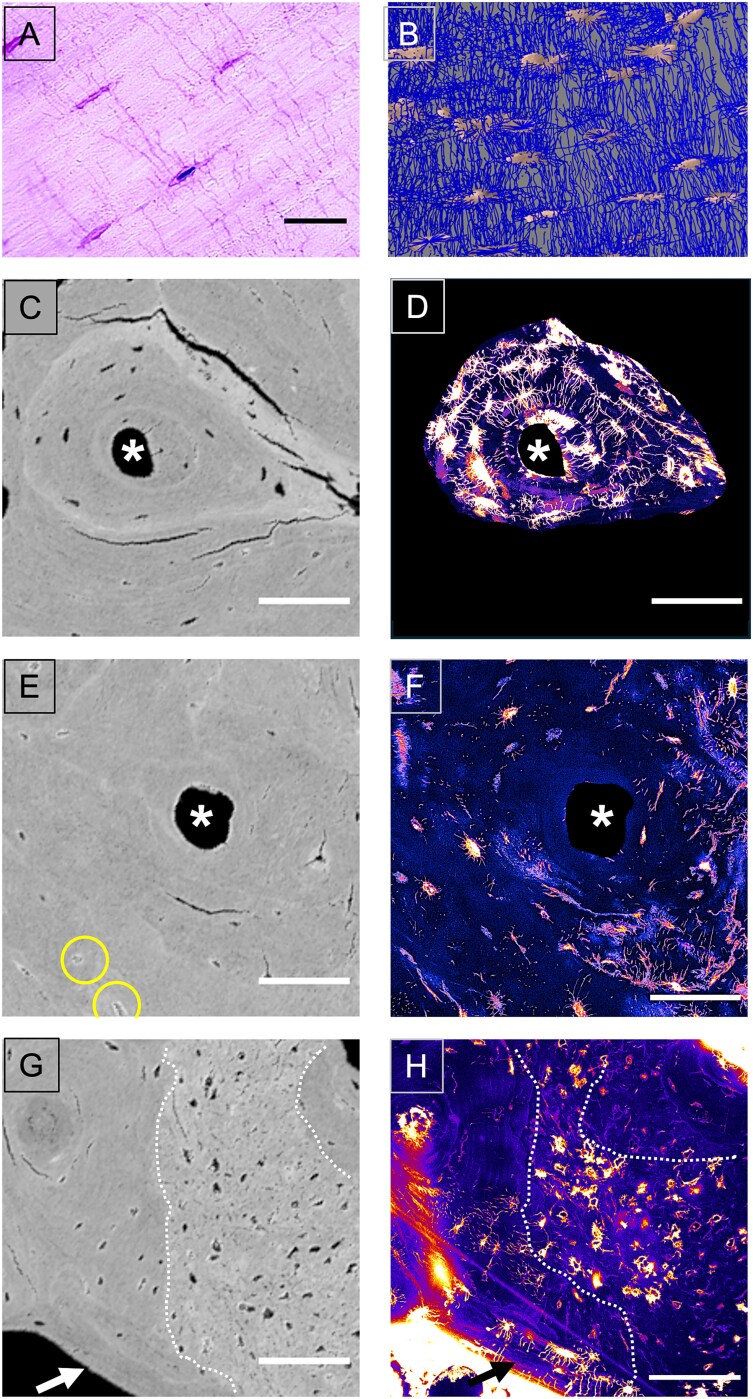
(A) Histological section from the parallel lamellar bone in ROI 3, showing osteocyte lacunae and canaliculi in histological sections, Giemsa staining and (B) the osteocyte lacuno-canalicular network (OLCN) in 3D rendering obtained with confocal microscopy from the same area (mean density = 0.055 μm/μm^3^). Note in (A) that osteocyte lacunae are elongated and oriented parallel to the bone lamellae, whereas canaliculi run perpendicularly to the long axis of the lacunae. Scalebar = 25 μm. (C-H) Same areas viewed by backscattered electron microscopy (B, D, and F) and by confocal laser scanning microscopy to image the OLCN stained by rhodamine (C, E, and G): (C and D): osteon with a well-developed, dense OLCN: osteocyte lacunae on B appear as dark (black) holes, and thus are not occluded and osteocyte lacunae and canaliculi are clearly visible on C. (E and F) Osteon with a distorted, OLCN: osteocyte lacunae on D are mostly micropetrotic and few osteocyte lacunae and canaliculi are viewed on E. The circles denote micropetrotic osteocytes. (G and H) “Patch” of woven bone within an area of lamellar bone: on the backscattered electron microscopy image F, woven bone appears as an area with elevated osteocyte lacunae density and brighter, thus higher mineralized than the surrounding bone tissue (broken line). By confocal laser scanning microscopy in G the osteocyte lacunae are clearly stained but the canalicular network is hardly visible. Note the difference to the osteocytes in the area of lamellar bone that exhibit ellipsoidal shape, and the canaliculi run perpendicularly to the long axis of the lacunae as shown in (A). The asterisks indicate haversian channels, arrows point to lamellar bone. Scalebars = 100 μm.

Osteocyte lacunae size and density were within normal reference range (see Table 2), but the aspect ratio was slightly decreased in trabecular bone and slightly increased in the cortical compartment. A smaller aspect ratio indicates that the osteocyte lacunae are more roundish, a typical feature of woven bone.^31^ In contrast, osteocytes in lamellar bone are known to reside in elliptical elongated lacunae (reflected by a higher aspect-ratio) that run parallel to the lamellae, with their canaliculi oriented perpendicular to the long axis of the lacunae and thus to the bone lamellae.^31^ This corresponds to what we observed in the cortical shell of our sample (Figure 5A and B).

### Micropetrosis leads to a local disruption of the OLCN

Figure 5C-H shows qBEI images of selected regions of the sample with corresponding images of the OLCN obtained from rhodamine fluorescence measured with the confocal microscope. Figure 5C and D demonstrate that in despite the absence of cathepsin K a regular OLCN is formed in regions of normal osteocyte lacunar density. In contrast, regions with a large number of micropetrotic lacunae exhibit only distorted networks with much lower canalicular density (Figure 5E and F). The network densities in these 2 regions were found to be 0.050 and 0.034 μm/μm^3^, respectively. The value of 0.050 μm/μm^3^ is close to the OLCN density measured in 3 other regions of the trabecular region of the sample giving 0.048 μm/μm^3^. This value is close to the trabecular OLCN density measured in 2 healthy reference samples yielding a value of 0.043 μm/μm^3^.

We further observed within “patches” of woven bone, irregularly shaped osteocyte lacunae that are unevenly distributed. In contrast to the situation shown in Figure 5F, the lacunae are not occluded (thus not micropetrotic) and therefore stained by the rhodamine dye. Nonetheless, their OLCN appears disrupted in the 2D plane because, typically for woven bone, the canaliculi radiate in all directions.^31^ As aforementioned, these disordered bone areas contrast markedly with the surrounding lamellar bone showing regularly distributed ellipsoid osteocyte lacunae and an ordered canalicular network (Figure 5G and H).

## Discussion

Recent reviews have delineated the natural history of pycnodysostosis and treatment of affected patients,^1^^,^^3^^,^^4^ but the alterations at the bone tissue level, in particular the effects leading to elevated bone fragility, are less well characterized. We present here bone material analyses from an adult female patient with short stature, open fontanelles, osteosclerosis, and a history of recurrent fractures with delayed healing from the first decade of life. She was diagnosed with pycnodysostosis, at the age of 50 yr, when she was finally referred to a specialized Bone Center at the Charité Berlin. Thus, during many years, nobody suspected nor recognized that she might suffer from a bone fragility disorder that warrants multidisciplinary clinical management. The performed genetic analyses revealed a *CTSK* variant (c.493C>T) (Figure 1), which is not found in common databases and, therefore, is extremely rare. Thus, it is surprising to find it in a homozygous state in an individual of German descent. No information of parental consanguinity was available. An alternative explanation is that 1 parent inherited a larger deletion encompassing the *CTSK* gene. At the time of analysis copy number analysis of NGS data was not yet available.

In accordance with the typical pycnodysostosis-associated osteosclerosis, our qBEI analyses revealed elevated trabecular volume and hypermineralization of the bone matrix in trabecular as well as in cortical bone (Figure 2). This concomitant increase in bone mass and matrix mineralization is in accordance with the well-accepted etiology of the disorder in which cathepsin K-deficient osteoclasts lead to a net decrease in bone resorption, whereas osteoblast-directed bone formation continues resulting in elevated bone mass.^32^ Hence, because bone tissue is less remodeled, the average tissue age and thus the average matrix mineralization is increased resulting in a stiffer and more brittle bone.^7^ Interestingly, in the outer cortical compartment we observed, by polarized light microscopy, multilayers of bone lamellae arranged in parallel to the periosteum mirroring robust periosteal bone apposition (Figure 4A-D). Such dense primary bone formation is indeed a characteristic of bone modeling that occurs independently of bone resorption^33^ and very likely contributes to the “thicker bone” phenotype observed in patients with pycnodysostosis as in murine models.^13^ In fact, elevated periosteal bone formation was reported in post-menopausal women treated with cathepsin K inhibitors^34^ but has not yet been demonstrated in individuals with cathepsin K deficiency. Although the underlying molecular mechanisms remain elusive, osteogenic periosteal stem cells are known to express cathepsin K^35^ and a recent study reported that inhibition or deletion of cathepsin K induces periosteal bone formation through activation of the Wnt-β-catenin pathway.^36^ It is, noteworthy that cathepsin K is also crucial for callus maturation and remodeling, and the lack of the enzyme does very likely contribute to impaired and/or delayed fracture healing and refracture in patients with pycnodysostosis.^37^ Although we did not analyze callus tissue, using polarized light microscopy we observed patches of disordered, randomly arranged collagen fibers, a typical feature of woven bone^31^ in the trabecular (Figure 3G and H) as well as in the cortical region between highly vascularized areas with well-developed haversian canals (Figure 4A, C, and D). Such woven bone inserts could further be identified on qBEI images as brighter, thus higher mineralized bone areas compared with the surrounding lamellar bone.^31^ Also the areas of woven bone exhibited dense and irregularly positioned roundish osteocyte lacunae (Figure 5G), in contrast to their ellipsoidal shape in lamellar bone (Figure 5A and B).^31^ Because woven bone is primarily a repair tissue also laid down during callus formation,^31^^,^^38^ it might represent a fingerprint of earlier, impaired fracture healing processes. However, we previously also noted chaotic lamellar arrangement in transiliac bone biopsy samples from young patients with pycnodysostosis^7^ and large amounts of disordered bone matrix was also observed in cathepsin K-deficient mice.^13^ Together, these observations suggest that the presence of poorly organized matrix is an intrinsic characteristic of bone tissue in pycnodysostosis, probably resulting from unbalanced bone remodeling.^15^ It is well accepted that woven or disordered lamellar bone is mechanically less competent than ordered lamellar bone.^38^ We and others have previously suggested that large inserts of disordered matrix interrupting the homogeneity of the mineralized matrix significantly contribute to the overall bone fragility.^7^^,^^13^^,^^31^ The present additionally study shows that within areas of woven bone, the OLCN is highly distorted (Figure 5H), suggesting impaired osteocyte function and decreased capacity to adapt to mechanical needs.

Typical histological features of pycnodysostosis are the large multinucleated, thus differentiated osteoclasts that are unable to degrade the demineralized matrix. From our light microscopy images, we cannot judge whether non-digested collagen or even mineralized matrix is phagocytosed by the osteoclasts as reported previously^6^ but we observed typical fringes of demineralized bone,^7^ which remained on the bone surface (Figure 3A-D). Unexpectedly, and in contrast to our previous observations in young adult pycnodysostosis patients showing only very few and flat shaped osteoclasts, indicative of reduced resorbing activity,^7^ in the present bone sample we observed a high number of large osteoclasts that were either attached (Figure 3C and D) or detached (Figure 3G and H) from the bone surfaces. Interestingly, we also noted in our patient areas with severe bone marrow fibrosis that has not been previously reported in *Ctsk-*deficient mice. Noteworthy, the fibrosis is very likely not secondary to hyperparathyroidism given that her PTH levels were within normal limits (Table S1), consistent with previous observations in patients with pycnodysostosis.^11^^,^^39^^,^^40^ Interestingly, bone marrow fibrosis has been previously observed in patients with so-called “osteoclast-rich” forms of osteopetrosis, thus, in forms of the disorder characterized by high number of dysfunctional osteoclasts due to mutations in genes encoding proteins involved in bone resorption.^30^ Hence, it seems that rather than the lack of cathepsin K itself, the elevated osteoclastogenesis in association with defective bone resorption leads somehow to alteration of collagen synthesis in marrow stroma.^30^ We can also only speculate on the reasons of such an abnormally high number of dysfunctional-osteoclasts in our patient. In our previous study, both investigated individuals carried homozygous missense mutations.^7^ Whether a loss of the cathepsin K protein due to nonsense mutations or impaired enzymatic activity due to missense mutations may differentially influence osteoclast numbers and morphology is currently unclear. Kiviranta et al.^10^ suggested that increasing numbers of osteoclasts might be a mechanism to compensate impaired bone resorption and demonstrated elevated osteoclastogenesis through RANKL/OPG ratio, an observation that was confirmed by additional studies.^12^^,^^15^ Chen et al.^41^ showed that osteoclast senescence is impaired in absence of cathepsin K, resulting in a prolonged lifetime of osteoclasts. We also noted many detached, very likely apoptotic osteoclasts (Figure 3G and H). These findings suggest that the ability to form high numbers of osteoclasts in pycnodysostosis does not only depend on age, as suggested previously,^7^ but possibly differ between skeletal sites, and might be boosted during to compensate incomplete remodeling processes during fracture healing.^37^

Within the last years an essential role of cathepsin K for osteocytic perilacunar remodeling essentially during lactation has been revealed.^15^ Of note, our patient was never pregnant and never lactated. Nevertheless, we were interested to examine whether the lack of the enzyme would impact the formation of the OLCN and osteocyte lacunar characteristics. The present findings indicate that cathepsin K is not essential the formation and maintenance of the OLCN although we cannot rule out a compensatory activity of other cathepsins or matrix metalloproteinases.^11^ Nonetheless, the density of the lacunar network (0.034-0.050 μm/μm^3^) was similar to the 2 age-matched reference bone samples from the iliac crest, but smaller than reported in cortical bone from the femoral midshaft in healthy individuals exhibiting OLCN densities around 0.07 μm/μm^3^.^42^ Clearly, more sample analyses are required to verify whether such differences represent intra- or interindividual variations or result from methodological differences. Moreover, qBEI evaluations showed that osteocyte lacunae size, shape, and density were within normal limits compared with age-matched reference values (Table 2).^28^ However, numerous bone packets exhibited highly elevated number of mineralized, thus, micropetrotic osteocyte lacunae (Table 2). Micropetrosis is a condition in which osteocyte lacunae and canaliculi fill in vivo with mineralized tissue following osteocyte death (recently reviewed by Milovanovic and Busse^43^). Elevated micropetrosis is associated with aging,^28^ and was demonstrated in patients with post-menopausal osteoporosis and further in metabolic bone disorders with elevated bone fragility.^43^ Most importantly, we did not only observe abnormally high levels of micropetrosis but also found that micropetrotic osteocyte lacunae were not randomly scattered throughout the bone matrix but seem to concentrate within specific bone packets (Figure 5C-F). In such bone packets, the osteocyte lacunar network was severely diminished indicating that the local disruptions of the OLCN throughout the bone matrix of our patient are in fact much more severe than one would expect only by the quantitative evaluation of the aforementioned patches of woven bone.

We further observed that the homogeneity of the bone matrix was interrupted by some irregularly shaped, calcified inclusions that we anticipated to be residual calcified cartilage (Figure 4E, G, and H). Mineralized cartilage remnants within the bone matrix are a hallmark of osteopetrosis^44^^,^^45^ and other forms of osteosclerosis^46^ and were also demonstrated within the bone tissue of a child with pycnodysostosis.^7^ Cartilage is generally higher mineralized than bone and therefore appears bright on backscattered electron microscopy images^47^ (Figures 2C and 4G) and can be identified as an additional shoulder on the right site of the BMDD curve,^46^ which we did however not observe in the bone sample from our patient (Figure 2D). One explanation is that the relative amount was small compared with the total amount of mineralized tissue evaluated within the ROI. EDX analyses confirmed the hydroxyapatite nature of the mineral component of these areas (calcium/phosphate ratio varied from 1.72 to 1.74 as in the adjacent mineralized bone matrix, see Table S2). However, we observed some striking features: we did not detect round chondrocytes and viewed a very low number of cells or osteocyte lacunae. Consistently, the EDX analyses showed highly reduced carbon, thus organic, content in these areas (Table S2). Moreover, by comparing the same feature in the light microscope by Goldner (Figure 4G) and Giemsa staining (Figure 4H), we noted that the calcified areas stained green for Goldner (which is atypical for mineralized cartilage that normally stains very pale by Goldner^48^) and purple for Giemsa. The latter could indicate the presence of proteoglycans, typically expected in cartilage but also for other acidic proteins. In the light of these uncertainties, we prefer designating these “macropetrotic” hypermineralized areas as dystrophic calcifications with uncertain etiology. It is nevertheless clear that the presence of dystrophic calcifications, or hypermineralized calcified cartilage remnants in the bone matrix, along with elevated micropetrosis and disordered bone matrix organization will again negatively impact the material properties of the bone tissue and therefore very likely contribute to a compromised bone strength and the overall bone fragility observed in pycnodysostosis.

In summary, our data indicate that in pycnodysostosis not only osteoclast function and bone turnover are impaired resulting in elevated bone mass with hypermineralization of the matrix, but also collagen organization is highly disturbed. Moreover, osteocyte viability is decreased leading to locally enhanced micropetrosis, distorted, and reduced canalicular networks and very heterogenous bone mineralization patterns. Thus, it seems that beyond osteoclast dysfunction, the lack of cathepsin K hampers also the ability of osteocytes to detect tissue damage and consequently to trigger repair processes. Most likely, the accumulation of areas with occluded, hypermineralized osteocyte lacunae will further affect bone material quality and contribute to bone fragility. Nevertheless, the observation of large areas of orderly and dense osteocyte network in our patient indicates that cathepsin K is not essential for the formation of the OLCN.

## Supplementary Material

Pycno_for_resubmission_Supplemental_Table_1_ziaf015

Pycno_ms_Supplemental_Table_2_for_resubmission_ziaf015

## Data Availability

Data supporting this publication can be accessed at our institutional digital data repository for published research via creed.lbg.ac.at.

## Notes

To the memory of Prof. Dieter Felsenberg, MD, Founder and former director of the Centre for Muscle and Bone Research at the Charité University, Campus Benjamin Franklin, Berlin, Germany.
