# Supplementary material for: Bone quality in pycnodysostosis: micropetrosis, locally distorted osteocyte lacuno-canalicular network, and heterogenous mineralization pattern in an adult female patient with multiple fractures
Source: JBMR Plus. 2025 Jan 23;9(4):ziaf015. doi: 10.1093/jbmrpl/ziaf015 (PMC11937824; doi:10.1093/jbmrpl/ziaf015)
Supplement: Pycno_for_resubmission_Supplemental_Table_1_ziaf015 [file pycno_for_resubmission_supplemental_table_1_ziaf015.docx]

**Supplemental Table 1**

Blood counts and bone turnover markers in the patient

| Parameter | Patient | Reference values |
| --- | --- | --- |
| Hemoglobin (g/dl) | 14.4 | 12 – 15.6 |
| Hematocrit | 0.432 | 0.355 – 0.455 |
| Erythrocytes (million/mm^3^) | 2.6 | 3.9 – 5.2 |
| Leukocytes (g/L) | 6.03 | 3.9 – 10.5 |
| Platelets (nb/nl) | 347 | 150 – 370 |
| Ca (mmol/l) | 2.59 | 2.15 – 2.50 |
| Phosphate (mmol/l) | 1.16 | 0.87 – 1.45 |
| 25-OH-Vitamin D3 (nmol/L) | 85.2 | 50 – 150 |
| PTH (pg/ml) | 22.9 | 15 – 65 |
| ALP (U/l) | 78 | 35 – 104 |
| Osteocalcin (μg/l) | 15.20 | 8.4 – 28 |
