# Supplementary material for: Bone quality in pycnodysostosis: micropetrosis, locally distorted osteocyte lacuno-canalicular network, and heterogenous mineralization pattern in an adult female patient with multiple fractures
Source: JBMR Plus. 2025 Jan 23;9(4):ziaf015. doi: 10.1093/jbmrpl/ziaf015 (PMC11937824; doi:10.1093/jbmrpl/ziaf015)
Supplement: Pycno_ms_Supplemental_Table_2_for_resubmission_ziaf015 [file pycno_ms_supplemental_table_2_for_resubmission_ziaf015.docx]

**Supplemental Table 2**: Results of EDX analyses of hypermineralized areas “macropetrosis” (MP) and adjacent mineralized bone matrix (AJMBM). Procession: Carbon by difference. All data are given in atomic %)

|  | Oxygen | Sodium | Magnesium | Calcium | Phosphate | Carbon | Ratio calcium/phosphate |
| --- | --- | --- | --- | --- | --- | --- | --- |
| MP1 | 64.65 | 1.26 | 0.28 | 20.62 | 11.98 | **1.21** | 1.72 |
| AJMBM 1 | 51.22 | 0.80 | 0.21 | 15.07 | 8.64 | **24.06** | 1.74 |
| MP2 | 61.91 | 1.13 | 0.29 | 20.03 | 11.60 | **5.05** | 1.73 |
| AJMBM 2 | 53.46 | 0.80 | 0.28 | 17.99 | 10.33 | **17.13** | 1.74 |

Note the low content of carbon in the areas of macropetrosis **(in bold)**
